# Genome-wide survey of cytochrome P450 genes in the salmon louse *Lepeophtheirus salmonis* (Krøyer, 1837)

**DOI:** 10.1186/s13071-019-3808-x

**Published:** 2019-11-27

**Authors:** Joseph L. Humble, Greta Carmona-Antoñanzas, Carol M. McNair, David R. Nelson, David I. Bassett, Ingibjørg Egholm, James E. Bron, Michaël Bekaert, Armin Sturm

**Affiliations:** 1 Institute of Aquaculture, University of Stirling, Stirling, FK9 4LA Scotland, UK; 2 Department of Microbiology, University of Tennessee, Memphis, TN 38163 USA

**Keywords:** Drug resistance, Aquaculture, Salmon farming, *Lepeophtheirus salmonis*, Caligidae, Cytochrome P450

## Abstract

**Background:**

The salmon louse (*Lepeophtheirus salmonis*) infests farmed and wild salmonid fishes, causing considerable economic damage to the salmon farming industry. Infestations of farmed salmon are controlled using a combination of non-medicinal approaches and veterinary drug treatments. While *L. salmonis* has developed resistance to most available salmon delousing agents, relatively little is known about the molecular mechanisms involved. Members of the cytochrome P450 (CYP) superfamily are typically monooxygenases, some of which are involved in the biosynthesis and metabolism of endogenous compounds, while others have central roles in the detoxification of xenobiotics. In terrestrial arthropods, insecticide resistance can be based on the enhanced expression of CYPs. The reported research aimed to characterise the CYP superfamily in *L. salmonis* and assess its potential roles in drug resistance.

**Methods:**

*Lepeophtheirus salmonis* CYPs were identified by homology searches of the genome and transcriptome of the parasite. CYP transcript abundance in drug susceptible and multi-resistant *L. salmonis* was assessed by quantitative reverse transcription PCR, taking into account both constitutive expression and expression in parasites exposed to sublethal levels of salmon delousing agents, ecdysteroids and environmental chemicals.

**Results:**

The above strategy led to the identification of 25 CYP genes/pseudogenes in *L. salmonis*, making its CYP superfamily the most compact characterised for any arthropod to date. *Lepeophtheirus salmonis* possesses homologues of a number of arthropod CYP genes with roles in ecdysteroid metabolism, such as the fruit fly genes *disembodied*, *shadow*, *shade*, *spook* and *Cyp18a1*. CYP transcript expression did not differ between one drug susceptible and one multi-resistant strain of *L. salmonis*. Exposure of *L. salmonis* to emamectin benzoate or deltamethrin caused the transcriptional upregulation of certain CYPs. In contrast, neither ecdysteroid nor benzo[a]pyrene exposure affected CYP transcription significantly.

**Conclusions:**

The parasite *L. salmonis* is demonstrated to possess the most compact CYP superfamily characterised for any arthropod to date. The complement of CYP genes in *L. salmonis* includes conserved CYP genes involved in ecdysteroid biosynthesis and metabolism, as well as drug-inducible CYP genes. The present study does not provide evidence for a role of CYP genes in the decreased susceptibility of the multiresistant parasite strain studied. 
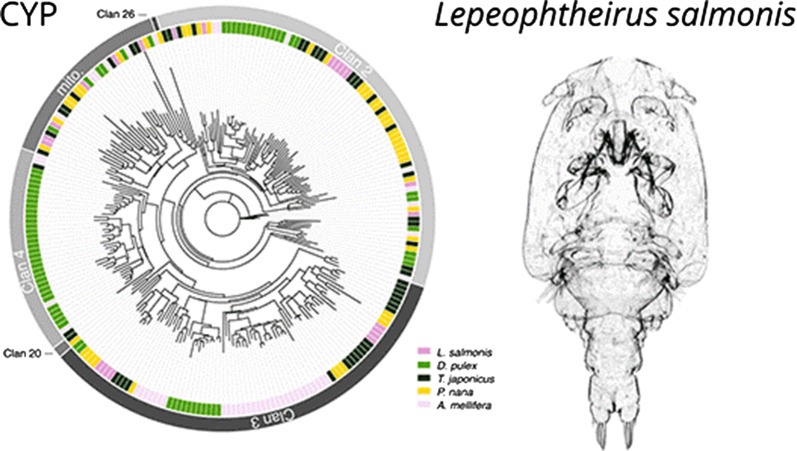

## Background

Caligid sea lice (Copepoda, Crustacea) are ectoparasites of marine fish, feeding on the mucus, skin and blood of their hosts [1]. The salmon louse, *Lepeophtheirus salmonis* (Krøyer, 1837) infests wild and farmed salmonid fishes and is divided into two allopatric subspecies, which inhabit the North Atlantic and the North-East Pacific [2]. Caligid infestations are a major obstacle to salmon farming due to their detrimental effects on the health and welfare of cage-cultured fish [3], to the economic costs associated with decreased yield and to the costs of parasite control measures [1, 4]. For 2017, global costs associated with sea louse infections and their control have been estimated at ~ £700 million [5]. In addition, concerns have been raised regarding the potential for detrimental impacts of transmission of farm-origin salmon lice to wild salmonid populations, which can be particularly vulnerable to salmon lice associated mortality at the migratory smolt phase of the anadromous life-cycle [6].

At salmon production sites, *L. salmonis* are managed using an integrated pest management strategy (IPM) employing a range of control approaches to maximize results. In the last five years, there has been an increased implementation of non-medicinal control strategies, which include co-culture with cleaner fish [7, 8], cage designs that reduce infection pressure [9], immunostimulants [10], treatments with freshwater and thermal delousing [11]. Despite this, *L. salmonis* control still relies significantly on a limited range of veterinary drugs [4]. Licensed salmon delousing agents currently available in the UK include the organophosphate azamethiphos, the pyrethroid deltamethrin (DM) and the non-specific oxidant hydrogen peroxide, all of which are applied as bath treatments, and the macrocyclic lactone emamectin benzoate (EMB), which is administered as an in-feed medication [12]. The repeated use of the same or similarly acting compounds can favour the evolution of resistance in parasite populations [13]. Loss of treatment efficacy has been reported for most available drugs [14–17], likely indicative of the development of drug resistance [18–21].

Resistance of terrestrial arthropod pests to insecticides and acaricides is relatively well understood and most commonly involves one or both of two main molecular mechanisms [22]. Resistance can be based on mutations causing specific amino acid substitutions in proteins targeted by the pesticide [23, 24]. Alternatively, resistance can result from enhanced detoxification due to overexpression of enzymes responsible for pesticide metabolism, which typically involves members of three large gene families, the cytochrome P450s (CYPs), carboxylesterases and glutathione transferases [25]. While resistance mechanisms in *L. salmonis* are still incompletely understood, recent studies provide first insights into the molecular determinants involved. *Lepeophtheirus salmonis* resistance to the organophosphate azamethiphos is a consequence of a point mutation in an acetylcholinesterase gene [18], whereas hydrogen peroxide resistance is associated with increased catalase expression [26]. Resistance of *L. salmonis* to EMB has been linked to selective sweeps; however, the genes under selection remain to be identified [27]. Pyrethroid resistance is mainly maternally inherited and associated with specific mitochondrial haplotypes [19], with possible additional roles of target site mutations in voltage gated sodium channels [20].

CYPs constitute a large gene superfamily of haem-containing enzymes present in prokaryotes and eukaryotes [28]. Metazoan CYPs are membrane-bound, locating either to the endoplasmic reticulum or to the inner mitochondrial membrane, and catalyse a diverse range of reactions related to the metabolism of endogenous and foreign chemicals [29]. The CYP superfamily shows high complexity, both between and within species, with the number of CYP loci in arthropod genomes ranging from 36 in the human body louse *Pediculus humanus humanus* to > 200 in the tick *Ixodes scapularis* [30, 31]. In insects, CYP superfamily members mediate steps in the biosynthesis of ecdysteroids [32], juvenile hormone [33] and cuticle long-chain hydrocarbons [34]. CYPs may further be involved in pheromone biosynthesis and odorant degradation at chemosensory sensilla [35]. Furthermore, a number of CYPs contribute to the biochemical defence against xenobiotics and are involved in the metabolic detoxification of phytotoxins [36] and pesticides [35].

In terrestrial arthropods, insecticide resistance can result from the constitutive upregulation of CYPs (reviewed in [35, 37, 38]), as first suggested by the overexpression of the *Cyp6g1* gene in DDT-resistant laboratory and field populations of *Drosophila melanogaster* [39]. For certain insect CYPs, roles as resistance factors have been corroborated by the demonstration of catalytic activity in the metabolism of relevant compounds [40, 41], protective effect of transgenic overexpression [42], and reversal of resistance by RNA interference [43, 44]. In *L. salmonis*, roles for CYPs in the toxicology of salmon delousing agents have been suggested based on changes in transcript expression of certain CYPs associated with resistance or previous exposure to treatments [45, 46]. However, the CYP superfamily of *L. salmonis* has not previously been annotated or systematically assessed regarding its potential roles in drug resistance.

The aim of the present study was to characterise the CYP superfamily in *L. salmonis* and to obtain insights into potential roles of CYPs in the resistance of this parasite against chemical control agents. CYPs were initially identified by homology searches of *L. salmonis* genome and transcriptome databases, and subsequently annotated and subjected to phylogenetic analyses. Constitutive transcript expression was compared, for CYPs identified in the *L. salmonis* transcriptome, between laboratory-cultured strains of multi-resistant and drug-susceptible parasites, and the effects of xenobiotic exposure on CYP transcription were also assessed.

## Methods

### Salmon lice husbandry

Laboratory-cultured strains of *L. salmonis* investigated in this study have previously been described [19, 47]. Strain IoA-00 is susceptible to all current salmon delousing agents, whereas strain IoA-02 has previously been shown to be resistant against EMB and DM. Azamethiphos susceptibility of the strains was determined in this study (see below). Since isolation, the strains have been cultured under identical conditions using Atlantic salmon (*Salmon salar* L.) as host, as described in detail previously [17, 47]. All experimental infections were conducted under UK Home Office licence, and were subject to prior ethical review and appropriate veterinary supervision. Prior to harvesting parasites for experimental use, salmon carrying sea lice were euthanized by percussive stunning followed by destruction of the brain, according to UK Home Office Schedule 1 requirements. Collected parasites were immediately placed into aerated filtered seawater equilibrated to 12 °C and allowed to recover for 2 to 6 h before being randomly allocated to experimental treatments.

### Chemical exposure experiments

*Lepeophtheirus salmonis* bioassays with azamethiphos (Salmosan Vet® 500, 50% w/w azamethiphos) were conducted at 12 °C and involved exposure of parasites to eight drug concentrations (0.46, 1.00, 2.15, 4.64, 10.0, 21.5, 46.4 and 100 µg/l) or seawater (controls). Drug and control treatments were run in duplicate, with each replicate containing each 5 adult males and 5 pre-adult-II or early adult females. Following 60 min of exposure, parasites were transferred to clean seawater and allowed to recover for 24 h before being rated as normal or impaired [18], using a set of behavioural criteria described in detail before [47]. Response data were assessed and the median effective concentration (EC_50_) derived by probit analysis using Minitab version 16.1.1.

In order to compare transcript expression between drug-susceptible and drug-resistant parasites and to further elucidate potential effects of different environmental and endogenous compounds on transcript abundance, male and female *L. salmonis* of the above strains were subjected to water-borne chemical exposures of chemicals at sublethal levels. Compounds studied included the salmon delousing agents EMB and DM, the arthropod hormones ecdysone (Ec) and 20-hydroxyecdysone (20HEc) and the environmental pollutant benzo[*a*] pyrene (BAP). All compounds studied were of analytical grade purity and obtained from Sigma-Aldrich (Dorset, UK).

PEG_300_ (polyethylene glycol, M_n_ =  300) was used to solubilise EMB and DM, while ethanol was used to solubilise E, 20HE and BAP. The final level of both solvents in treatments and controls was 0.05% (v/v). No effects of PEG_300_ on transcript expression were detected in a previous microarray study [45]. Exposure solutions (EMB: 25 and 150 µg/l; DM: 0.05 and 2 µg/l; Ec and 20HEc: 0.02 and 0.2 µg/l; BAP: 0.003 and 0.03 µg/l) were prepared using filtered seawater. Reflecting recommended conditions for immersion bath treatments, *L. salmonis* were exposed to DM for 30 min, followed by the transfer of animals to clean seawater and 24 h of recovery. Exposure to EMB and all other compounds were for 24 h. After exposure and (if applicable) recovery, the viability of parasites was confirmed by ascertaining the absence of behavioural responses defined for bioassays [47], prior to removal of parasites into RNA stabilisation solution (4.54 M ammonium sulphate, 25 mM trisodium citrate, 20 mM EDTA, pH 5.4). Samples were stored overnight at 4 °C, before transfer to nuclease-free tubes for storage at − 80 °C pending RNA extraction.

### Identification and annotation of *L. salmonis* CYP genes

In order to identify *L. salmonis* CYP sequences, a previously published multi-stage *L. salmonis* transcriptome (EBI ENA reference ERS237607) [48] was screened for CYP genes by conducting parallel tBLASTn searches (cut-off E-value of 10^−5^), employing as query sequences the full complement of CYP proteins of different arthropods in which the CYP gene superfamily has been annotated (*Tigriopus japonicus* [49], *Paracyclopina nana* [50], *Daphnia pulex* [51], *Drosophila melanogaster* [52]). *Daphnia pulex* sequences were obtained from supplementary materials available in the online version of a previous study [51]; see Additional file 1: Table S1 for accession numbers of sequences). The *L. salmonis* genome assembly LSalAtl2s (metazoa.ensembl.org) was scanned for CYP sequences using the same strategy, with query sequences further including CYP transcripts identified in the *L. salmonis* transcriptome. Each CYP locus identified by the above strategies was manually annotated following the criteria of the Cytochrome P450 Nomenclature Committee. The CYP superfamily is subdivided into CYP families containing members of > 40% amino acid identity, and subfamilies comprised of sequences of at least 55% amino acid identity [53]. CYP names consist of the superfamily designation ‘CYP’ followed by a number denoting the family and a letter indicating the subfamily, plus a final number attributed to the isoform. *Lepeophtheirus salmonis* CYP sequences identified and named as described above were confirmed by RT-PCR and sequencing experiments (see below) and deposited in GenBank (see Additional file 2: Table S2 for accession numbers).

### Phylogenetic analyses

CYPs from *L. salmonis* (this study) were subjected to phylogenetic analyses together with CYPs from three crustaceans (*D. pulex* [51], *T. japonicus* [49], *P. nana* [50]) and one insect (honeybee, *Apis mellifera* [54]) (see Additional file 1: Table S1 for accession numbers). Peptide sequences were aligned using GramAlign v3.0 [55] and analysed using IQ-TREE v1.6.9 [56]. The phylogenetic tree was constructed using a maximum likelihood method implementing the GTR model for heterogeneity among sites and the Dayhoff substitution model [-m Dayhoff+G8+FO] with 1000 bootstrapping iterations [-bb 1000].

### RNA extraction

Individual *L. salmonis* were homogenised in 500 µl TriReagent (Sigma-Aldrich) using a bead-beater homogenizer (BioSpec, Bartlesville, Oklahoma, USA) and total RNA was extracted by following the manufacturer’s protocols. RNA was resuspended in MilliQ water (20 µl for females and 15 µl for males). RNA purity and concentration was inspected by spectrophotometry using a NanoDrop ND-1000 (Thermo Fisher Scientific, Paisley, UK) and the values for the 260 nm/280 nm ratio were recorded as within the range of 2.0–2.3, while RNA integrity was assessed by following electrophoresis on horizontal agarose gels and visualization of ethidium bromide-stained bands under UV light.

### cDNA synthesis

Total RNA samples were reverse transcribed using BioScript Reverse Transcriptase (Bioline, London, UK) following the manufacturer’s protocols. RNA (300 ng) was combined with anchored oligodT (1 µM, Eurofins Genomics, Ebersberg, Germany) and random hexamers (3 µM, Qiagen, Manchester, UK), 1 µM of dNTPs and nuclease-free water in a volume of 10 µl. Following incubation at 70 °C for 5 min and cooling on ice for 5 min, each reaction aliquot received 4 µl RT buffer, 1 µl RiboSafe Inhibitor, 1 µl of BioScript reverse transcriptase, 1 µl DTT (20 mM) and 3 µl nuclease-free water. The reactions were then incubated at 25 °C for 10 min, 42 °C for 30 min and 85 °C for 5 min. In addition to samples, negative controls were included that lacked reverse transcriptase. Products were stored at − 20 °C.

### RT-PCR and sequencing

In order to confirm *L. salmonis* CYP sequences identified in this study, cDNAs were amplified by reverse transcription polymerase chain reaction (RT-PCR) and sequenced (see Additional file 2: Table S2 for primer sequences). PCR reactions were conducted using the Q5® Hot Start High-Fidelity 2× Master Mix (New England Biolabs, Hitchin, UK) following the manufacturer’s protocol and employing 35 cycles. PCR products were examined by agarose gel electrophoresis and the remaining PCR product was purified (QIAquick PCR Purification Kit, Qiagen) and submitted to a commercial provider for Sanger sequencing. Sequences obtained for the same PCR products were aligned to obtain contiguous cDNA sequences (Table 1), which were deposed in GenBank (see Additional file 2: Table S2 for accession numbers).

**Table 1:** The *Lepeophtheirus salmonis* CYP superfamily. CYPs were identified by homology searches in transcriptome (EBI ENA reference ERS237607) and genome assemblies (LSalAtl2s, ensemble.metazoa.org) and annotated following the criteria of the Cytochrome P450 Nomenclature Committee

*Lepeophtheirus salmonis* CYP sequence					Annotation				
P450 clan	CYP name	Length (aa)	Transcript	Gene	Best BLAST hit	Accession number	Species	E-value	Identity (%)
CYP2	CYP18P1	527	HACA01008353^a^	EMLSAG00000004688	Cytochrome P450 18E1	AKH03496.1	*Paracyclopina nana*	0.00E+00	53.56
CYP2	CYP307N1	476	HACA01014463^a,b^; HACA01014464^b^	EMLSAG00000001150^b^	Cytochrome P450 307F1	AKH03498.1	*Paracyclopina nana*	5.00E−121	45.09
CYP2	CYP3031C1	526	HACA01022487^a^	EMLSAG00000005163	Cytochrome P450 CYP3031A1	AIL94135.1	*Tigriopus japonicus*	2.00E−124	40.00
CYP2	CYP3038E1	548	HACA01006511^b^	EMLSAG00000005721^a^	Cytochrome P450 CYP3038B1	APH81379.1	*Tigriopus kingsejongensis*	5.00E−139	42.09
CYP2	CYP3041C1	490	HACA01003809^a^	EMLSAG00000007328	Cytochrome P450 3041B1	AKH03506.1	*Paracyclopina nana*	2.00E−145	48.92
CYP2	CYP3041C2	480	HACA01027076^b^; HACA01031477^b^	EMLSAG00000002359^a^	Cytochrome P450 3041B1	AKH03506.1	*Paracyclopina nana*	9.00E−145	46.30
CYP2	CYP3041D1	481	HACA01029496^a^	EMLSAG00000007758	Cytochrome P450 CYP3041A2	AIL94133.1	*Tigriopus japonicus*	3.00E−123	42.65
CYP2	CYP3041E1	477	HACA01001994^a^; HACA01011887	EMLSAG00000007334^b;^ EMLSAG00000007335^b^; EMLSAG00000011475^b^	Cytochrome P450 CYP3041A2	APH81382.1	*Tigriopus kingsejongensis*	9.00E−126	40.57
CYP2	CYP3041E2	482	HACA01000555^a^	EMLSAG00000006822	Cytochrome P450 3041B1	AKH03506.1	*Paracyclopina nana*	2.00E−118	41.19
CYP3	CYP3027H1	484	HACA01003852^a,c^	EMLSAG00000010829	Cytochrome P450 3A24	ACO15001.1	*Caligus clemensi*	0.00E+00	65.45
CYP3	CYP3027H2	482	HACA01014781^a^	EMLSAG00000009405	Cytochrome P450 3A24	ACO15001.1	*Caligus clemensi*	0.00E+00	63.73
CYP3	CYP3027H3	482	HACA01004583^a^	EMLSAG00000005269	Cytochrome P450 3A24	ACO15001.1	*Caligus clemensi*	0.00E+00	63.09
CYP3	CYP3027H4	494	HACA01012946^b^	EMLSAG00000012088^a^	Cytochrome P450 3A24	ACO15001.1	*Caligus clemensi*	0.00E+00	62.31
CYP3	CYP3027H–fragment1	197	–	EMLSAG00000010833^a,b^	Cytochrome P450 3A24	ACO10681.1	*Caligus rogercresseyi*	5.00E−33	64.52
CYP3	CYP3027H–fragment2	87	–	EMLSAG00000006848^a,b^	Cytochrome P450 3A24	ACO10681.1	*Caligus rogercresseyi*	7.00E−26	66.20
CYP3	CYP3649A1	537	HACA01001887^a,b^	EMLSAG00000004666^b^	Cytochrome P450 CYP3025B1	APH81387.1	*Tigriopus kingsejongensis*	3.00E−111	36.08
CYP3	CYP3649A2	537	HACA01004064^a^	EMLSAG00000006058	Cytochrome P450 CYP3025B1	APH81387.1	*Tigriopus kingsejongensis*	2.00E−122	36.02
CYP3	CYP3649A–fragment1	107	–	EMLSAG00000002804^a,b^	Cytochrome P450-like protein 3	ADB28828.1	*Tigriopus japonicus*	1.00E−08	41.18
CYP3	CYP3651A1P	492	HACA01014825^a^	–	Cytochrome P450 CYP3025B1	APH81387.1	*Tigriopus kingsejongensis*	8.00E−22	23.72
Mitochondrial	CYP44M1	483	HACA01005509^a^	EMLSAG00000008058	Cytochrome P450 CYP44D1	APH81396.1	*Tigriopus kingsejongensis*	4.00E−144	44.05
Mitochondrial	CYP44M2	431	HACA01005507^a,b^	EMLSAG00000008058	Cytochrome P450 CYP44D1	APH81396.1	*Tigriopus kingsejongensis*	7.00E−126	44.34
Mitochondrial	CYP302A1	470	HACA01015112^b^; HACA01015113^a^	EMLSAG00000005374	Putative ecdysteroid 22-hydroxylase	EFX63066.1	*Daphnia pulex*	1.00E-144	47.50
Mitochondrial	CYP314A1	527	HACA01031265^a^	EMLSAG00000009224	Cytochrome P450 CYP314A1	AIL94172.1	*Tigriopus japonicus*	0.00E+00	54.46
Mitochondrial	CYP315E1	421	–	EMLSAG00000003403^a,e^	Cytochrome P450 315A1	AKH03535.1	*Paracyclopina nana*	6.00E−85	38.00
Mitochondrial	CYP3650A1	478	HACA01009722^a^	EMLSAG00000005044	Cytochrome P450 3020B1	AKH03536.1	*Paracyclopina nana*	7.00E−95	36.75

RT-PCR followed by Sanger sequencing was used to confirm cDNA sequences, which were deposited in GenBank (see Additional file 2: Table S2 for accession numbers)

^a^Predicted polypeptide length based on this sequence

^b^Partial sequence

^c^HACA01003852 contains a one-base deletion disrupting the open reading frame, predicted peptide length according to corrected sequence based on RT-PCR/sequencing data

^d^HACA01015113 contains a one-base deletion disrupting the open reading frame, predicted peptide length according to corrected sequence based on RT-PCR/sequencing data

^e^Gene model EMLSAG00000003403 is the fusion between a CYP gene and a kinase, probably reflecting an assembly problem. Polypeptide length based on CYP sequence only

### RT-qPCR

Quantitative reverse transcription polymerase chain reaction (RT-qPCR) was used to determine the transcript abundance of CYP sequences identified in the transcriptome. Six male and six female parasites were analysed for each combination of treatment and strain. Primers were designed using primer-BLAST (NCBI) to anneal to, or surround, intron-exon boundaries when available. Primers for target and reference genes (ribosomal subunit 40S, *40S*; elongation factor 1-alpha, *ef1a*; and hypoxanthine-guanine phosphoribosyltransferase, *hgprt*) [45] (Additional file 3: Table S3) were used at 300 µM with 2.5 µl of a 1:20 dilution of the cDNA synthesis reaction with Luminaris Color HiGreen qPCR Master Mix (Thermo Fisher Scientific) in a total volume of 10 µl. Reactions were performed in technical duplicate for experimental samples and technical triplicate for standard curve, non-template controls and reverse transcriptase controls in a LightCycler 480 II (Roche Diagnostics, Basel, Switzerland) using white 384-well plates. The thermocycling program (95 °C for 10 min, then 40 cycles of 95 °C for 15 s, 60 °C for 30 s, 72 °C for 30 s, then 72 °C for 3 min) was followed by melting curve analysis. Relative transcript quantification was achieved by including on each PCR plate a set of serial dilutions of a pool of all experimental cDNA samples, allowing derivation of the estimated relative copy number of the transcript of interest for each sample, this being corrected for the efficiency of the reaction (Additional file 4: Table S4). The normalized expression values were generated by the ΔΔCt method [57] and the results expressed as mean normalized ratios between the relative units of each target gene and a reference gene index calculated from the geometric mean of the threshold cycles of the three reference genes.

### Statistical analyses

As a number of RT-qPCR data sets failed tests of homoscedasticity (Minitab version 17), non-parametric tests were used for statistical analysis of the data. All further tests were conducted in R version 3.4.1, using the packages *rcompanion* and *PMCMR*. The Scheirer-Ray-Hare test was used to assess effects of parasite strain and sex/stage on transcript expression. The Kruskal–Wallis test was employed to check for effects of chemical treatments. To control the experiment-wise type I error, sequential Bonferroni correction was applied to account for the simultaneous testing of 21 transcripts [58]. Following significant Kruskal–Wallis results, Dunn’s test was used for *post-hoc* comparisons between chemical treatments to the control group.

## Results

### Identification of *L. salmonis* CYPs

In order to identify CYPs in *L. salmonis*, homology searches were carried out in a previously published multi-stage transcriptome [48] and a genome assembly (LSalAtl2, metazoan.ensembl.org) of the parasite. In the transcriptome, 25 sequences were identified, all of which except for transcript HACA01014825 could be mapped to gene models of the genome assembly, with some gene models being represented by more than one transcript (Table 1). Transcript HACA01014825 showed signs of pseudogenisation such as multiple in-frame stop codons, mapped to a genome region in supercontig LSalAtl2s111 lacking a gene model. Homology searches of the genome assembly for CYP sequences yielded four further potential CYP loci, three of which were short partial sequences. Taken together, 25 putative CYP genes/pseudogenes were obtained in *L. salmonis* and named according to the current CYP nomenclature (Table 1). Alignment and assessment of the sequences revealed the conservation of motifs present in arthropod CYPs, namely the helix C, helix I, helix K, PERF and haem binding motifs (Additional file 5: Table S5).

### Phylogenetic analyses

**Fig. 1:**
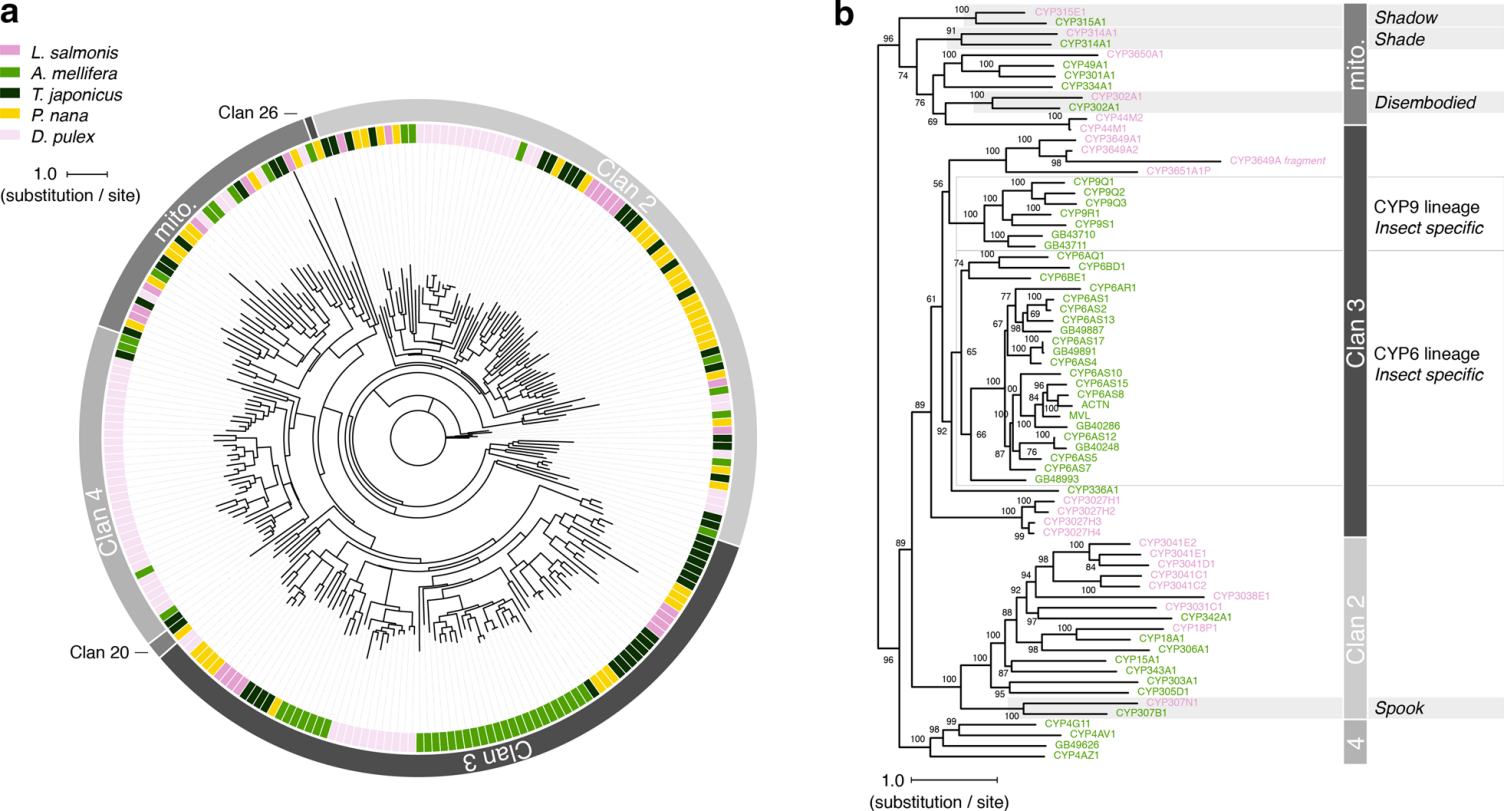
Phylogenetic analysis of 22 *L. salmonis* CYPs. Peptide sequences were aligned using the programme GramAlign v3.0 [54] and analysed using IQ-TREE v1.6.9 [55]. **a** Phylogenetic tree of CYPs from *L. salmonis* and four arthropod species (*Daphnia pulex*, *Tigriopus japonicus*, *Paracyclopina nana* and *Apis mellifera*). **b** Phylogenetic tree of CYPs from *L. salmonis* and *A. mellifera*. Numbers at the branching points of nodes represent percent bootstrap support values

*Lepeophtheirus salmonis* CYPs were subjected to phylogenetic analysis together with sequences from crustaceans in which the CYP superfamily has been characterised, i.e. the branchiopod *Daphnia pulex* [51] and the non-parasitic copepods *Tigriopus japonicus* and *Paracyclopina nana* [49, 50] (Fig. 1a). Salmon louse CYPs were further analysed regarding their evolutionary relation to CYPs of the honeybee (*Apis mellifera)* [54] (Fig. 1b). Both analyses differentiated CYP clans as distinct clades, with *L. salmonis* sequences found within the mitochondrial CYP, CYP2 and CYP3 clans. The phylogenetic analysis further suggested that *L. salmonis* possesses homologues of a number of insect Halloween genes encoding CYPs involved in ecdysteroid biosynthesis, including *spook*/CYP307A1, *disembodied*/CYP302A1, *shadow*/CYP315A1 and *shade*/CYP314A1 but not *phantom*/CYP306A1 (Fig. 1b). Furthermore, *L. salmonis* appeared to possess a homologue of CYP18A1, a 26-hydroxylase functioning in ecdysteroid inactivation.

### Transcript expression of *L. salmonis* CYPs

**Fig. 2:**
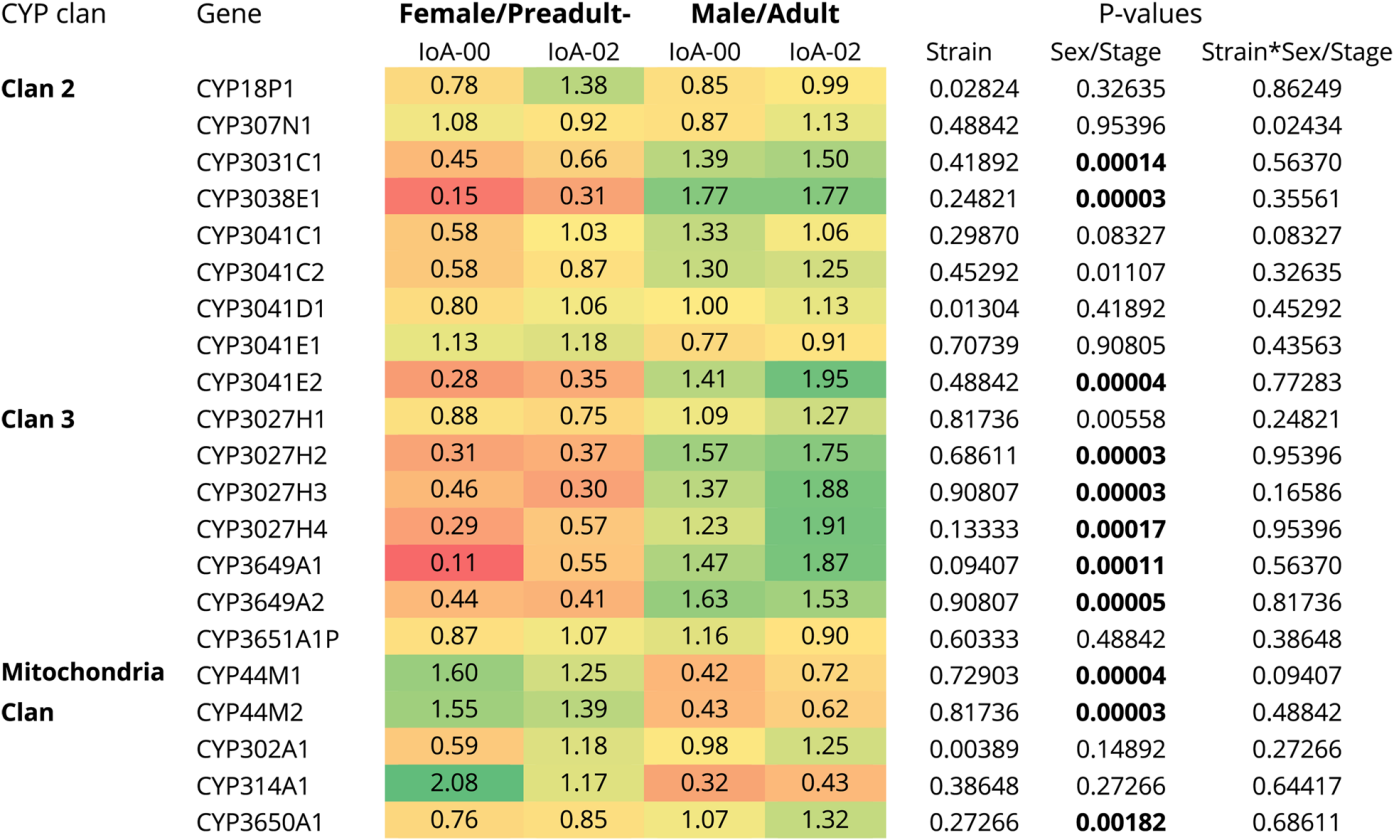
CYP transcript expression in two *L. salmonis* strains. Transcript expression of CYPs was determined by RT-qPCR in preadult-II females and adult males of two *L. salmonis* strains (IoA-00: drug-susceptible, IoA-02: multi-resistant). The transcript abundance in the relevant stage and strain is expressed as fold expression compared to the average expression among all groups, with above average expression highlighted in green and below average expression in red. Effects of strain, sex/stage and interaction of strain and sex/stage were assessed by the Scheirer-Ray-Hare test. *P*-values significant after Bonferroni correction are given in bold print

The transcript expression of *L. salmonis* CYPs was studied using quantitative real-time PCR (RT-qPCR) in two previously characterised laboratory-maintained strains of the parasite. Strain IoA-00 is susceptible to all licensed chemical salmon delousing agents, whereas strain IoA-02 is resistant against EMB, DM and azamethiphos (Additional file 4: Table S4). CYP transcript expression was studied in synchronised parasite cohorts of developmental stages typically used for immobility bioassays (male adult, female preadult-II). Eleven of 21 studied CYP transcripts, including representatives from all clans, were differentially expressed between male adult and female preadult-II lice (Fig. 2). In contrast, differences in CYP transcription between the two strains investigated were not significant (Fig. 2).

**Fig. 3:**
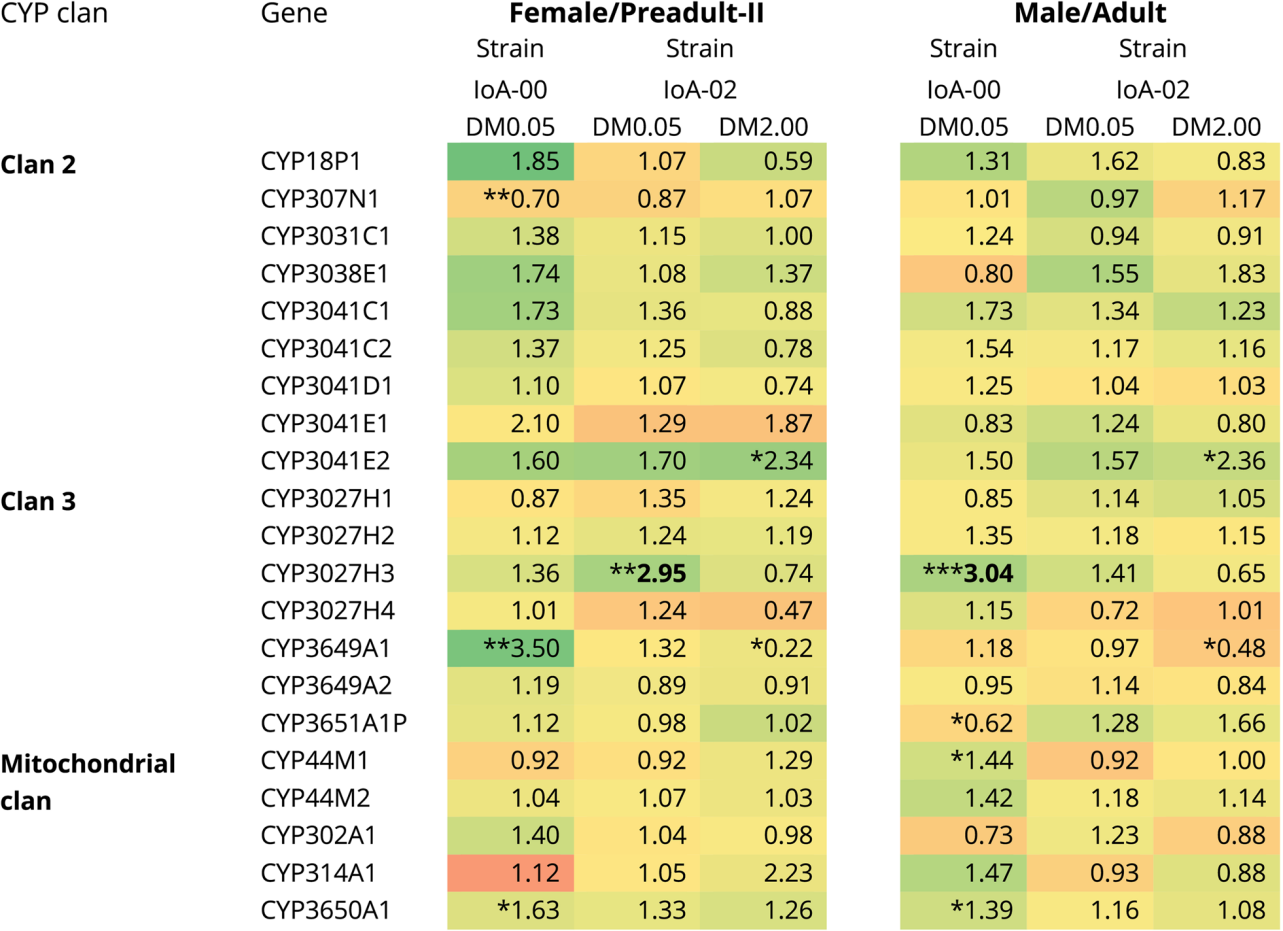
Effects of deltamethrin on CYP transcript expression in *L. salmonis*. Preadult-II females and adult males of two *L. salmonis* strains (IoA-00, drug-susceptible; IoA-02, multiresistant) were exposed to deltamethrin (DM0.05, 0.05 µg/l; DM2.00, 2.0 µg/l) for 30 min and allowed to recover for 24 h in clean seawater before CYP transcript abundance was determined by RT-qPCR. Transcript levels in exposed parasites are given as fold expression compared to untreated control animals, with upregulation highlighted in green and downregulation in red. Data were subjected to Kruskal–Wallis tests (bold: significant after Bonferroni correction) followed by *post-hoc* comparisons to the control group (Dunn’s test; *P < 0.05, **P < 0.01, ***P < 0.001)

**Fig. 4:**
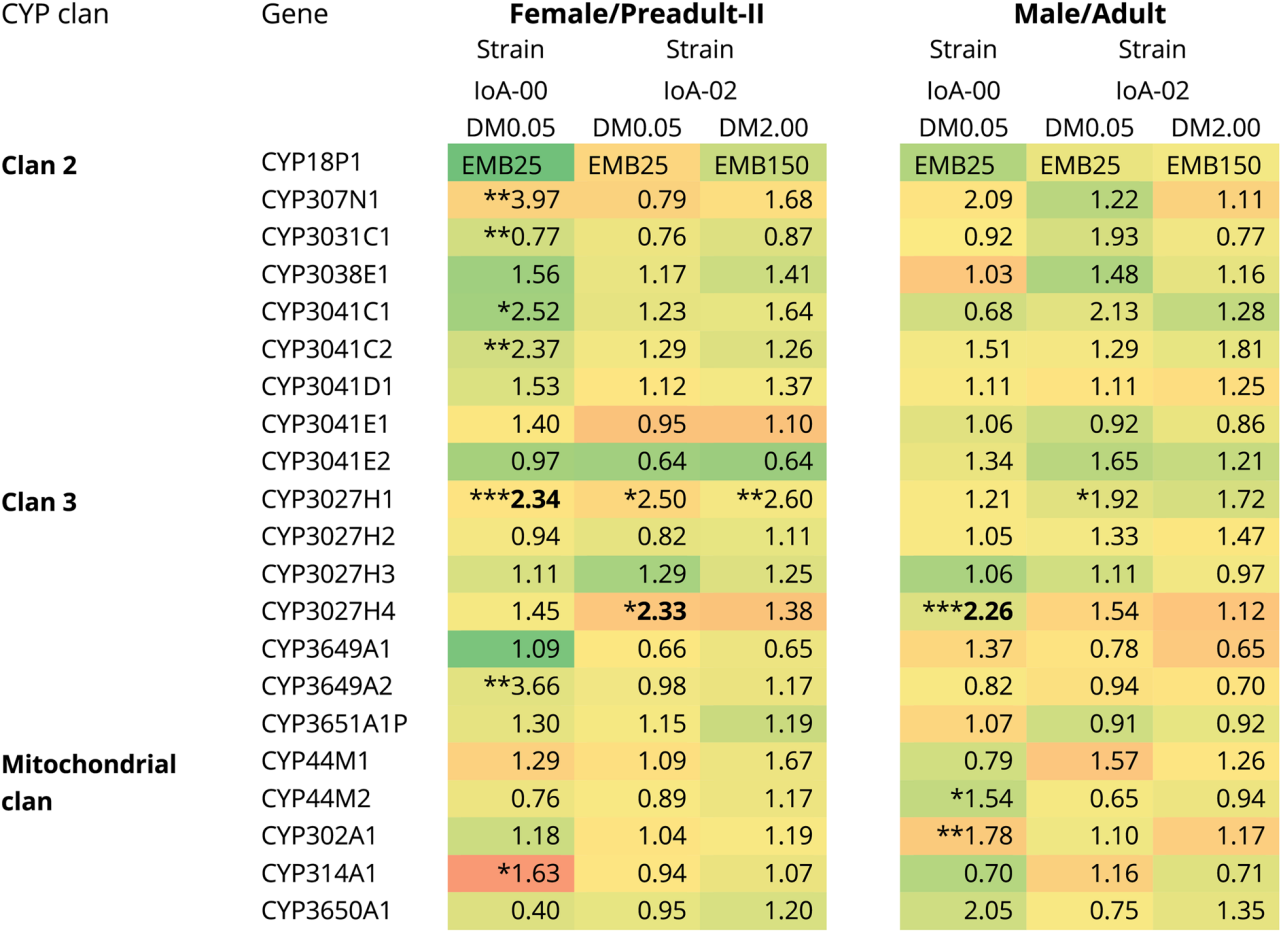
Effects of emamectin benzoate (EMB) on CYP transcript expression in *L. salmonis*. Preadult-II females and adult males of two *L. salmonis* strains (IoA-00, drug-susceptible; IoA-02, multiresistant) were exposed to emamectin benzoate (EMB25, 25 µg/l; EMB150, 150 µg/l) for 24 h before CYP transcript abundance was determined by RT-qPCR. Transcript levels in exposed parasites are given as fold expression compared to untreated control animals, with upregulation highlighted in green and downregulation in red. Data were subjected to Kruskal-Wallis tests (bold: significant after Bonferroni correction) followed by *post-hoc* comparisons to the control group (Dunn’s test; *P < 0.05, **P < 0.01, ***P < 0.001)

The effects of drugs on CYP transcription were studied for two salmon delousing agents, the pyrethroid DM (Fig. 3) and the macrocyclic lactone EMB (Fig. 4). Experiments involved the exposure of IoA-00 and IoA-02 lice to low sublethal concentrations of the compounds (0.05 µg/l DM; 25 µg/l EMB) and both strains were exposed to higher concentrations (2.0 µg/l DM, 150 µg/l EMB). The latter were sublethal to strain IoA-02, allowing studies of transcript expression, but as expected lethal to IoA-00 (data not shown), with no surviving parasites available for expression studies. Compared to transcript levels in control parasites, treatments with both 0.05 µg/l DM and 25 µg/l EMB caused upregulation of CYP3027H3 in IoA-00 adult males and IoA-02 preadult-II females (Figs. 3, 4). Moreover, an increased transcript abundance of CYP3041E2 was observed in IoA-00 preadult-II females after exposure to 25 µg/l EMB (Fig. 4).

Finally, the effects of the ecdysteroids Ec and 20HEc and the environmental chemical BAP on CYP transcription were investigated in the IoA-02 strain. No significant effects on CYP transcript abundances were observed in the experiment (Additional file 6: Figure S1).

## Discussion

The present report provides the first genome-wide survey of the CYP superfamily in *L. salmonis,* a parasite causing considerable economic costs in aquaculture [5]. In the North Atlantic, *L. salmonis* has developed resistance against most chemical control agents [21], including the pyrethroid DM [19, 59] and the macrocyclic lactone EMB [17, 60]. In terrestrial arthropod pests, resistance to pyrethroids [61, 62] and macrocyclic lactones [63, 64] can be based on the enhanced expression of CYPs involved in pesticide metabolism. The present study did not find evidence for altered CYP transcript expression in a multiresistant *L. salmonis* strain as compared to a drug-susceptible reference strain. However, in both strains, certain CYPs were upregulated following sublethal drug exposures.

The eukaryotic CYP superfamily is highly diverse, showing complexity within and between species. Molecular phylogenetic analyses of animal CYPs have identified 11 deep-branching clades called CYP clans [29], ten of which emerged early in metazoan evolution [65]. Clan losses occurred in the lineage leading to insects, in which the CYP superfamily is composed of four clans (mitochondrial, CYP2, CYP3 and CYP4). The 25 putative CYP genes/pseudogenes identified in *L. salmonis* in this study could be unequivocally assigned to the mitochondrial, CYP2 and CYP3 clans, which are also present in other crustaceans such as the branchiopod *D. pulex* [51], isopods [66] and the copepods *T. japonicus* and *P. nana* [49, 50]. However, while *L. salmonis* and *P. nana* show an apparent lack of CYP4, this clan is present in other crustaceans [49, 51, 66]. Moreover, the free-living copepods *T. japonicus* and *P. nana* possess CYP20 genes, with *P.* *nana* additionally having one CYP26 clan gene [49, 50]. The CYP20 and CYP26 clans are present in cnidarians [65], suggesting their emergence prior to the divergence of bilaterians. CYP20 and CYP26 genes have been retained in chordates and lophotrochozoans [67], as well as some arthropods such as chelicerates and myriapods [66], but were lost in lineages leading to insects and most crustacean groups.

The CYP superfamily includes genes that can be classified as environmental response genes [68], as they encode proteins involved in interactions external to the organism. Examples are the CYPs involved in pesticide resistance, which show characteristic traits of environmental response genes such high diversity, frequent expansion by duplication events and fast rates of evolution [69]. In contrast, CYPs involved in the biosynthesis of endogenous compounds such as hormones commonly show conservation between species. In insects, members of the CYP15 family encode epoxidases involved in juvenile hormone biosynthesis, with some differences between insect orders regarding the late steps of juvenile hormone biosynthesis and the chemical identity of the principal juvenile hormone [70]. In crustaceans, juvenile hormone biosynthesis lacks an epoxidation step and methyl farnesoate performs a similar role to juvenile hormone [71]. The failure to identify CYP15 members in *L. salmonis* (this study) is in line with the absence of this CYP family in crustaceans studied so far, such as *D. pulex* [51], *T. japonicus, P. nana* [49, 50] and *Neocaridina articulata* [72].

Ecdysteroids are key arthropod hormones with a variety of physiological roles, including the regulation of moulting [73, 74]. In insects and crustaceans, the most important ecdysteroids are Ec and 20HEc. The biosynthesis of these ecdysteroids involves a set of CYPs called the Halloween genes, originally identified in fruit fly [32]. After the conversion of cholesterol of dietary origin to 7-dehydrocholesterol by the Rieske-like oxygenase *neverland*, halloween genes catalyse the remaining steps of ecdysteroid biosynthesis. The first of these steps, still poorly understood and referred to as “black box” reactions, involves two CYP307 family paralogues in *Drosophila*, *spook* (CYP307A1) and *spookier* CYP307A2. Other insects may possess a further paralogue, spookiest (CYP307B1) believed to have a similar role. The remaining Halloween genes, *phantom* (CYP306A1), *disembodied* (CYP302A1) and *shadow* (CYP315A1) and *shade* (CYP314A1) are hydroxylases modifying the ecdysteroid at the 25-, 22-, 2- and 20-positions, respectively. Further related to ecdysteroid metabolism is CYP18A1 [75], a 26-hydroxylase inactivating the bioactive steroid 20-hydroxyecdysone.

*Lepeophtheirus salmonis* orthologues of *neverland*, *disembodied* and *shade* have recently been reported and characterised regarding their tissue distribution [76]. The present study further identified putative *L. salmonis* homologues of *spook/spookier* (CYP307A1/2), *shadow* (CYP315A1) and a CYP18A1 homologue. The failure of genome and transcriptome scans of this study to identify a *L. salmonis* homologue of *phantom* could be either due to absence of this gene in *L. salmonis*, or lack of its representation in current sequence repositories. *Phantom* is lacking in chelicerates [71], in which ponasterone A (25-deoxy-20-hydroxyecdysone) likely represents the bioactive ecdysteroid [77]. Arguing against a lack of *phantom* in *L. salmonis*, Ec, 20HEc and ponasterone A have been reported in larval and female adult stages of the parasite [76], with the biosynthesis of the former two hormones requiring 25-hydroxylase activity [78].

Compared to the number of CYP genes in free-living crustaceans, e.g. 75 in the phyllopod *D. pulex* [51] and 52 and 46 in the non-parasitic copepods *T. japonicus* and *P. nana* [49, 50], respectively, the *L. salmonis* CYP superfamily appears very small. A reduction in the size of gene superfamilies with roles in the biochemical defence against xenobiotics has previously been reported from insect ectoparasites lacking free-living stages, such as the human body louse (37 CYPs), compared to non-parasitic insects such as the fruit fly (85 CYPs) or ectoparasites possessing free-living life stages such as mosquitoes (204 CYPs) [30, 79]. Direct exposure to environmental toxins for such species may be reduced as a result of their parasitic lifestyle, with biochemical detoxification pathways of the host providing further protection. Supporting this hypothesis, previous studies of the ABC (ATP-binding cassette) gene family, which encodes membrane transporters many of which function in the detoxification of xenobiotics and endogenous compounds, found that *L. salmonis* possesses only 33 ABC genes [48], compared to 64 members of this gene superfamily in *D. pulex* [80].

In the present study, 11 of 21 studied CYPs differed significantly in transcript expression between preadult-II females and adult males. These stages were selected for study as they appear at the same time in synchronised cohorts of developing parasites and have approximately the same size and are well defined physiologically, whereas the large adult females undergo significant post-moulting growth and cycles of egg production and vitellogenesis [81], making this stage heterogeneous. While the moulting cycle can strongly affect CYP expression in crustaceans [82, 83], as can be expected for CYPs involved in ecdysteroid biosynthesis and metabolism, *L. salmonis* halloween genes and CYP18P1 were not found to be differentially expressed between preadult-II females and adult males in this study. Sex-biased transcript expression of CYPs in *L. salmonis* has previously been described from a microarray study, which included 12 CYPs, of which six showed sex-biased transcription [84].

In the present study, differences in CYP transcript expression between the multiresistant strain IoA-02 and the drug-susceptible reference strain IoA-00 were not significant. However, exposure to both DM and EMB caused significant transcriptional upregulation of CYP3027H3 in IoA-02 females and IoA-00 males, with EMB exposure further increasing CYP3041E2 transcription in IoA-00 females. In a previous microarray study [46], effects of the pyrethroid cypermethrin on transcript expression in *L. salmonis* copepodids included 3.8-fold upregulation of CYP3027H4 (referred to as “CYP3A24”, GenBank: JP326960.1) and 5.3- to 7.9-fold upregulation of CYP3649A2 (represented twice and referred to as “CYP6w1” or “CYP6d4”, GenBank: JP317875.1 and JP334550.1). Moreover, transcripts of CYP3031C1 and CYP3041C2, referred to by BLAST annotations as “CYP18A1” and “CYP15A1”, have been found to be constitutively overexpressed in an EMB resistant *L. salmonis* strain in an earlier microarray study [45]. Taken together, the data from this study and previous microarray studies suggest that a number of *L. salmonis* CYPs, particularly in clans CYP2 and CYP3, have roles as environmental response genes. Support for such roles of the CYP3027 family is provided by studies with free-living copepods *T. japonicus* [49] and *P. nana* [50], in which members of families CYP3027 and CYP2024 were transcriptionally upregulated following crude oil exposure. Interestingly, signature sequences typical for genes with roles in the detoxification of chemicals, such as aryl hydrocarbon responsive elements, xenobiotic responsive elements and metal response elements, were found in the promotor regions of oil-responsive *T. japonicus* CYPs [49].

## Conclusions

The CYP superfamily of *L. salmonis* is the smallest of all arthropods characterised to date. *Lepeophtheirus salmonis* CYPs include conserved genes involved in ecdysteroid biosynthesis and metabolism, as well as drug-inducible genes. In the parasite strains studied, no evidence was found for a role of CYP genes in mediating drug resistance.

## Supplementary information

**Additional file 1: Table S1.** Accession numbers of arthropod CYPs.

**Additional file 2: Table S2.** Oligonucleotide primer sequences used in RT-PCR and sequencing experiments.

**Additional file 3: Table S3.** Oligonucleotide primer sequences used in RT-qPCR experiments.

**Additional file 4: Table S4.** Susceptibility of *L.* *salmonis* strains to salmon delousing agents.

**Additional file 5: Table S5.** Conserved motifs in *L.* *salmonis* CYP predicted amino acid sequences.

**Additional file 6: Figure S1.** Effects of ecdysteroids and benzo[a]pyrene on *L. salmonis* CYP transcript expression.

## Data Availability

The raw datasets for RT-qPCR analyses and bioassays used in the present study are available from the corresponding author upon request. All other data generated or analysed during this study are included in this published article and its additional files.

## Abbreviations

CYP: cytochrome P450
PCR: polymerase chain reaction
RT-PCR: reverse transcription PCR
RT-qPCR: quantitative RT-PCR
IPM: integrated pest management
DDT: dichlorodiphenyltrichloroethane
EC_50_: median effective concentration
EMB: emamectin benzoate
DM: deltamethrin
Ec: ecdysone
20HEc: 20-hydroxyecdysone
BaP: benzo[*a*]pyrene
PEG: polyethylene glycol
Mn: number average molar mass
EDTA: ethylenediaminetetraacetic acid
cDNA: complementary DNA
