# Supplementary material for: Genome-wide survey of cytochrome P450 genes in the salmon louse Lepeophtheirus salmonis (Krøyer, 1837)
Source: Parasit Vectors. 2019 Nov 27;12:563. doi: 10.1186/s13071-019-3808-x (PMC6880348; doi:10.1186/s13071-019-3808-x)
Supplement: Supplementary file 4 — Additional file 4: Table S4. Susceptibility of L. salmonis strains to salmon delousing agents. [file 13071_2019_3808_MOESM4_ESM.docx]

**Additional file 4: Table S4.** Susceptibility of *Lepeophtheirus salmonis* strains to different salmon delousing agents. Results are expressed as median effective concentrations (EC_50_).

|  | EC_50_ (95% confidence intervals)  (µg L^-1^) | | | |
| --- | --- | --- | --- | --- |
| Strain | Emamectin benzoate^1^ |  | Deltamethrin^2^ | Azamethiphos^3^ |
|  | Females | Males | Mixed sex^4^ | Mixed sex^4^ |
|  |  |  |  |  |
| IoA-00 | 78.6 (70.0 – 88.3) | 92.4 (83.2 – 106.7) | 0.28 (0.28 – 0.36) | 2.76 (2.29 – 3.54) |
| IoA-02 | 335.3 (277.0 – 409.4) | 675.0 (618.0 – 738.0) | 40.1 (22.1 – 158.9) | 63.7 (60.2 – 127.5) |
| Resistance ratio^5^ | 4.3 | 7.3 | 143 | 23 |

^1^Data from reference [47].

^2^Data from reference [19].

^3^Data from this study.

^4^Dose-response curves for females and males did not differ significantly (P>0.05).

^5^Resistance ratio = EC_50_ (IoA-02) / EC_50_ (IoA0-00)
