# Supplementary material for: Genome-wide survey of cytochrome P450 genes in the salmon louse Lepeophtheirus salmonis (Krøyer, 1837)
Source: Parasit Vectors. 2019 Nov 27;12:563. doi: 10.1186/s13071-019-3808-x (PMC6880348; doi:10.1186/s13071-019-3808-x)
Supplement: Supplementary file 5 — Additional file 5: Table S5. Conserved motifs in L. salmonis CYP predicted amino acid sequences. [file 13071_2019_3808_MOESM5_ESM.docx]

**Additional file 5: Table S5.** **Conservation of CYP motifs in *L. salmonis* CYP sequences.** Amino acids conforming to the consensus sequence of the motif are given in bold print.

Helix C Helix I Helix K PERF Heme binding

WxxxR GxE/DTT/S ExLR PxxFxPE/DRF PFxxGxRxCxG/A

CYP18P1 **W**KTQ**R** SV**ETT** **E**IM**R** **P**EE**F**R**PERF** **PF**GV**G**Q**R**K**C**L**G**

CYP307N1 LQEE**R** **G**HSAV **E**TV**R** **P**LNYN**P**K**RF** **PF**SI**G**R**R**S**C**V**G**

CYP3031C1 **W**KQQ**R** **G**S**ETT** **E**IQ**R** **P**DQ**F**N**PDRF** **PF**SI**G**K**R**S**C**P**G**

CYP3038E1 **W**KFL**R** **G**A**ETS** **E**VR**R** **P**DI**F**N**P**S**RF** **PF**GF**G**R**R**R**C**I**G**

CYP3041C1 **W**QTQ**R** **G**A**ETS** **E**VQ**R** **P**LE**F**K**PERF** **PF**GL**G**R**R**I**C**M**G**

CYP3041C2 **W**KEQ**R** **G**G**ETS** **E**IQ**R** **P**KS**F**R**PERF** **PF**GV**G**K**R**I**C**M**G**

CYP3041D1 **W**QSQ**R** **G**S**ETT** **E**VQ**R** **P**HE**F**N**P**Y**RF** **P**YGF**G**K**R**I**C**M**G**

CYP3041E1 **W**QTQ**R** **G**S**ETS** **E**IQ**R** **P**QE**F**N**P**L**RF** **PF**GF**G**K**R**I**C**M**G**

CYP3041E2 **W**QNQ**R** **G**A**ETT** **E**IQ**R** **P**QQ**F**N**PDR**W **PF**GF**G**K**R**I**C**M**G**

CYP3027H1 **W**KLL**R** **G**YA**TT** **E**T**LR** **P**EI**F**K**PERF** **PF**GD**G**N**R**I**C**I**A**

CYP3027H2 **W**KLL**R** **G**YV**TT** **E**A**LR** **P**EE**F**K**PERF** **PF**GD**G**N**R**I**C**I**A**

CYP3027H3 **W**KLL**R** **G**YS**TT** **E**T**LR** **P**NE**F**K**PERF** **PF**GD**G**N**R**S**C**I**A**

CYP3027H4 **W**KLL**R** **G**YS**TT** **E**T**LR** **P**NE**F**K**PERF** **PF**GN**G**N**R**S**C**I**A**

CYP3027H-fragment1 ----- ----- ---- --------- **PF**GD**G**N**R**I**C**I**A**

CYP3027H-fragment2 ----- ----- **E**A**LR** **P**EELK**PERF** -----------

CYP3649A1 **W**KKI**R** **G**F**DTT** **E**V**LR** **P**EV**F**N**P**NN**F** A**F**GQ**G**P**R**N**C**I**G**

CYP3649A2 **W**KSI**R** **G**F**ETT** **E**V**LR** **P**TK**F**N**P**NN**F** A**F**GH**G**P**R**N**C**I**G**

CYP3649A-fragment1 ----- EYSKK **E**V**LR** --------- -----------

CYP3651A1P **W**NTFT YF**ETT** **E**IVE --------- -----------

CYP44M1 **W**WKL**R** **G**LS**TT** **E**T**LR** **P**LIHK**PER**W **PF**SH**G**T**R**M**C**I**G**

CYP44M2 **W**WKL**R** **G**LS**TT** **E**TF**R** **P**LIHK**PER**W **PF**SK**G**T**R**M**C**V**G**

CYP302A1 **W**WRI**R** **G**I**DTS** **E**T**LR** **P**HV**F**N**P**K**R**W **PF**GF**G**P**R**M**C**I**G**

CYP314A1 **W**HTL**R** **G**V**DT**I **E**SF**R** AKE**F**I**PER**W **PF**GF**G**K**R**I**C**P**G**

CYP315E1 **W**LRM**R** AV**DTT** **E**AS**R** **P**EH**F**L**P**Q**R**W **PF**GFRA**R**S**C**I**G**

CYP3650A1 **W**YDF**R** **G**V**DT**A **E**V**LR** **P**LE**F**R**PER**Y **PF**GY**G**P**R**V**C**V**G**
