# Supplementary material for: Genome-wide survey of cytochrome P450 genes in the salmon louse Lepeophtheirus salmonis (Krøyer, 1837)
Source: Parasit Vectors. 2019 Nov 27;12:563. doi: 10.1186/s13071-019-3808-x (PMC6880348; doi:10.1186/s13071-019-3808-x)
Supplement: Supplementary file 6 — Additional file 6: Figure S1. Effects of ecdysteroids and benzo[a]pyrene on L. salmonis CYP transcript expression. [file 13071_2019_3808_MOESM6_ESM.pdf]

Additional File 6: Figure S1. Effects of model inducers on CYP transcript expression in *L. salmonis*. Preadult-II females and adult males of multiresistant *L. salmonis* strain IoA-02 were exposed different levels of ecdysteroids (Ec: ecdysone, 20HEc: 20-hydroxy-ecdysone) or benzo[a]pyrene (BaP) for 24 h before before CYP transcript abundance was determined by RT-qPCR. Transcript levels in exposed parasites are given as fold expression compared to untreated control animals, with upregulation highlighted in green and downregulation in red. Data were subjected to Kruskal-Wallis tests (none significant after Bonferroni correction) followed by post-hoc comparisons to the control group (Dunn's test; *** P<0.001, ** P<0.01, *P<0.05).

Preadult-II females

		Ec		20HEc		BaP	
		0.2 µg L^{-1}	2.0 µg L^{-1}	0.2 µg L^{-1}	2.0 µg L^{-1}	0.003 µg L^{-1}	0.03 µg L^{-1}
Clan 2	CYP18P1	1.65	*2.59	*3.22	2.15	1.32	*2.76
	CYP307N1	*1.76	1.43	**1.90	1.65	1.20	*1.89
	CYP3031C1	0.86	0.85	1.02	1.47	1.11	0.99
	CYP3038E1	2.52	0.76	0.86	1.64	0.91	2.03
	CYP3041C1	1.15	1.04	1.00	1.28	1.33	1.32
	CYP3041C2	1.08	1.07	1.05	1.40	1.41	1.39
	CYP3041D1	1.19	1.14	1.08	1.22	1.50	1.21
	CYP3041E1	0.91	0.77	0.67	0.53	0.62	0.49
	CYP3041E2	2.37	1.07	0.89	1.96	1.22	1.80
Clan 3	CYP3027H1	0.99	0.72	0.67	1.01	0.70	0.89
	CYP3027H2	1.77	1.07	0.97	1.82	1.16	1.76
	CYP3027H3	1.25	1.00	0.62	1.41	0.85	1.55
	CYP3027H4	1.17	0.74	0.40	1.36	0.98	1.05
	CYP3649A1	1.09	1.07	0.82	1.50	1.16	1.37
	CYP3649A2	1.79	1.02	0.99	1.78	1.26	1.64
	CYP3651A1P	1.21	1.30	1.22	1.35	1.51	1.40
Mitochondrial clan	CYP44M1	0.78	0.89	1.11	1.21	0.96	0.63
	CYP44M2	0.91	1.18	1.18	1.04	1.10	0.89
	CYP302A1	0.88	0.78	0.81	0.92	0.81	0.85
	CYP314A1	0.65	0.89	1.27	0.44	0.90	1.29
	CYP3650A1	0.94	0.82	0.68	0.83	0.88	0.81

Adult males

		Ec		20HEc		BaP	
		0.2 µg L^{-1}	2.0 µg L^{-1}	0.2 µg L^{-1}	2.0 µg L^{-1}	0.003 µg L^{-1}	0.03 µg L^{-1}
Clan 2	CYP18P1	1.98	*2.96	*2.62	*3.07	**3.25	1.67
	CYP307N1	0.78	0.79	0.83	0.75	0.62	0.75
	CYP3031C1	1.08	0.93	1.50	1.28	0.86	0.75
	CYP3038E1	0.95	1.55	0.71	1.00	1.05	1.88
	CYP3041C1	1.11	0.88	1.31	1.09	1.22	1.25
	CYP3041C2	1.47	1.21	1.62	1.46	1.27	1.30
	CYP3041D1	0.99	0.92	1.22	1.18	1.07	0.90
	CYP3041E1	1.57	*2.41	1.77	2.08	**2.89	**2.63
	CYP3041E2	1.18	1.85	1.09	1.48	1.72	**3.27
Clan 3	CYP3027H1	1.04	1.22	1.10	1.43	1.24	1.32
	CYP3027H2	1.42	1.51	1.71	1.84	1.68	1.56
	CYP3027H3	1.48	1.12	1.60	1.55	1.51	1.35
	CYP3027H4	1.02	0.75	1.01	1.20	1.34	0.99
	CYP3649A1	0.53	0.73	1.20	0.99	0.61	0.88
	CYP3649A2	1.42	1.38	1.37	1.63	1.22	1.33
	CYP3651A1P	1.09	1.18	0.90	1.27	1.14	1.40
Mitochondrial clan	CYP44M1	0.92	1.38	1.10	1.06	1.35	1.06
	CYP44M2	1.15	1.40	1.22	1.25	*1.49	1.45
	CYP302A1	1.03	0.94	0.81	0.90	0.73	1.39
	CYP314A1	1.01	0.72	1.33	1.49	1.76	1.62
	CYP3650A1	1.09	1.21	1.39	1.33	1.54	1.14
